# Using circulating reproductive hormones for sex determination of Atlantic sturgeon (*Acipenser oxyrinchus oxyrinchus*) in the Saco River estuary, Maine

**DOI:** 10.1093/conphys/cow059

**Published:** 2016-12-09

**Authors:** Carolyn R. Wheeler, Ashleigh J. Novak, Gail S. Wippelhauser, James A. Sulikowski

**Affiliations:** 1 Department of Marine Sciences, University of New England, 11 Hills Beach Road, Biddeford, ME 04005, USA; 2 Maine Department of Marine Resources, 172 State House Station, Augusta, ME 04333, USA

**Keywords:** Atlantic sturgeon, non-lethal sampling, sex determination, steroid hormones

## Abstract

The Atlantic sturgeon (*Acipenser oxyrinchus oxyrinchus*) is a long-lived, anadromous fish species ranging from Labrador, CA to Florida, USA. In the Saco River, located in the Gulf of Maine, this species was not present during a survey study ending in 1982, but was found inhabiting the estuary in 2007. Although the reason for the return of this sturgeon to this river system remains unknown, research on basic life-history information is necessary to facilitate the conservation of this federally protected species. Given the conservation status of the species, the present study used circulating sex steroid hormones to determine the sex of 288 Atlantic sturgeon captured between 2012 and 2014 in the Saco River estuary located in the Gulf of Maine. Overall, the sex was determined for 93% of Atlantic sturgeon sampled. Mean hormone values were similar to other Atlantic sturgeon reproductive studies. The findings indicate the validity of sex steroid hormones as a singular method for sex determination in wild Atlantic sturgeon. Results also indicated a likely 1:1 (male:female) sex ratio in the system, except in 2014 when a 1:3 ratio was observed. It is not believed that the Saco River estuary is used for spawning, as several impassable dams block access to spawning habitat. However, this area might provide crucial foraging for growth and development of juveniles and a habitat for adults forgoing spawning.

## Abstract (precis)

The reproductive hormones associated with sex have been well studied in many sturgeon species. Here, these hormones are quantified as a non-lethal method to determine the sex and maturity of Atlantic sturgeon. The findings imply that the study area is used by multiple life stages, providing evidence for the importance of the habitat.

## Introduction

The Atlantic sturgeon (*Acipenser oxyrinchus oxyrinchus*) is a large, k-selected, anadromous fish ranging from Labrador, CA to Florida, USA [Atlantic sturgeon Status Review Team (ASSRT), 2007]. This species was highly prized for its caviar and flesh, initiating a robust fishery for Atlantic sturgeon, peaking at 3350 metric tons in 1870 (Smith and Clungston, 1997; Secor and Waldman, 1999). However by 1901, the fishery was harvesting only 10% of its peak levels largely owing to the species’ aforementioned life-history traits (Smith and Clungston, 1997). After ongoing declines in the population, the fishery was finally terminated in 1998 (Atlantic States Marine Fisheries Commission, 1998). While commercial targeting of Atlantic sturgeon is currently prohibited (Atlantic States Marine Fisheries Commission, 1990), populations are threatened by other factors, such as habitat loss from damming (i.e. blocking passage to spawning grounds; ASSRT, 2007), poor water conditions (i.e. decreasing dissolved oxygen concentrations from pollutants; Niklitschek and Secor, 2005) and incidental catch in other fisheries (ASSRT, 2007). Given this and the failure of populations to rebound despite a prohibited status, Atlantic sturgeon were listed in 2012 under the federal Endangered Species Act (ESA). This listing designated populations in the Gulf of Maine (GOM) Distinct Population Segments (DPS) as threatened and DPS units in the southern extent of the range (New York Bight, Chesapeake Bay, Carolina and Southern Atlantic) as endangered (ASSRT, 2007).

An essential component for assessing and managing fish populations is determining species reproductive parameters, such as when sexual maturity occurs, the timing of seasonal cycles and locations of spawning aggregations (e.g. Walker, 2005). These data are important for outputs such as generating population models used to estimate sustainable harvest levels and assessing risk of extinction or recovery by the species (Walker, 2005). However, these types of data are poorly characterized for the majority of systems inhabited by Atlantic sturgeon because of their large size, high mobility, depleted stock status, undetermined sex-specific genes and lack of external sexual dimorphism (Scott and Crossman, 1973; ASSRT, 2007; Keyvanshokooh and Gharaei, 2010). Thus, the acquisition of this information has largely been through lethal or sublethal techniques, such as gross dissections (e.g. Barannikova *et al*., 2004) and endoscopy to view and collect gonadal tissue for histological analysis (e.g. Petochi *et al*., 2011). Although these techniques are widely accepted and implemented in aquaculture practices, they are far less suitable for wild populations that are severely depleted or endangered (Boreman, 1997; Kynard and Kiiffer, 2002; Colombo *et al*., 2004). Thus, given the stock status and recent listing of the species, the development and use of effective non-lethal methods for Atlantic sturgeon are needed to inform management decisions without further compromising the population of this protected species.

Previous studies have successfully used circulating reproductive hormones [i.e. testosterone (T) and 17β-estradiol (E_2_)] to identify sex and reproductive status in captive (e.g. Amiri *et al*.,1996a, b; Semenkova *et al*., 2002; Barannikova *et al*., 2004; Davail-Cuisset *et al*., 2011) and wild sturgeon (e.g. Van Eenennaam *et al*., 1996; Ceapa *et al*., 2002; Webb *et al*., 2002; Heise *et al*., 2009). For example, T is an important androgen contributing to maturation in males and females (Cuisset *et al*., 1995; Moberg *et al*., 1995; Van Eenennaam *et al*., 1996; Amiri *et al*., 1996a, b; Webb *et al*., 2002), whereas E_2_ has been shown to increase during vitellogenesis and decrease during the terminal stages of oocyte maturation in females (Amiri *et al*., 1996b; Doroshov *et al*., 1997; Webb *et al*., 2002). Thus, by using a ratio of T and E_2_ concentrations, the sex of sturgeon can be determined (Craig *et al*., 2009). For example, significant differences in androgen and E_2_ concentrations were observed between sex and reproductive phases (i.e. ovulating, gravid and spent females) in Atlantic sturgeon sampled within the Hudson River (Van Eenennaam *et al*., 1996). In addition, Heise *et al*. (2009) identified the sex of Gulf sturgeon (*Acipenser oxyrinchus desotoi*) by E_2_ concentrations that clustered into a low and high group (<0.5 and >8 ng ml^−1^, respectively). Likewise, Craig *et al*. (2009) found it possible to use these reproductive hormones to identify sex in lake sturgeon (*Acipenser fulvescens*), but noted the need for a species-specific analysis for more accurate results. Finally, many hormone-based studies have been paired with other sex-determining methods (i.e. gross dissection and endoscopy) to valid hormone profiles (Webb *et al*., 2002). These data collectively have given rise to sets of accurate functions (up to 95% accurate) using T and E_2_ concentrations to determine sex (Webb *et al*., 2002), providing analysis techniques for future fully non-lethal studies.

For Atlantic sturgeon, reproductive research has typically been conducted within large river systems with documented spawning populations, such as the Hudson (Van Eenennaam *et al*., 1996; Kahnle *et al*., 1998, 2005) and Delaware Rivers (Secor and Waldman, 1999; Secor, 2002). Watersheds where access to freshwater habitat is limited tend to be overlooked because they are not thought to hold reproductive significance for this species (ASSRT, 2007). The Saco River, located in the GOM DPS, is the fourth largest river in Maine (Furey and Sulikowski, 2011); however, it is not considered an historical spawning site for Atlantic sturgeon because of the impassable Cataract Falls and a dam at river kilometre (rkm) 10 constructed about 1682 (ASSRT, 2007; US Army Corps of Engineers, 2013), which limits access to freshwater. As a result of heavy fishing pressures within the GOM near the turn of the 20th century (Atlantic States Marine Fisheries Commission, 1990), Atlantic sturgeon were not present in the Saco River Estuary (SRE) during a survey study from 1979 to 1982 (Reynolds and Casterlin, 1985). However, after a considerable absence from the SRE, an Atlantic sturgeon was captured in 2007 during a routine trawl survey (Furey and Sulikowski, 2011). This finding initiated a study aiming to investigate the possible resurgence of the species. Gillnet sampling conducted by Little (2013) from 2008 to 2011 resulted in an mean catch per unit effort of 7.26 Atlantic sturgeon per hour, comparable to that reported for the Kennebec system for the period 1998–2000 (ASSRT, 2007; catch per unit effort = 7.43). This high catch per unit effort and the limited available life-history information created an opportunity to use circulating steroid sex hormones to assess the reproductive status and sex ratio of Atlantic sturgeon captured within the SRE. Simultaneously, the importance of the system to the recovery of the species could be assessed.

## Materials and methods

### Capture

Atlantic sturgeon were collected between May and November from 2012 to 2014. Sampling was conducted primarily in the mouth of the SRE between the two jetties that extend ~2.3 rkm into Saco Bay (Brothers *et al*., 2008). Specifically, sampling occurred with bottom gillnets (15.2 or 30.5 cm stretched mesh × 2 m height × 100 m long) fished at slack low tide perpendicular to the jetties for 15 min. Following capture, fish were carefully removed from the gillnet and transferred to a net-pen (2.1 m × 0.9 m × 0.9 m), which was submerged in river water reflecting the ambient condition of the SRE.

### Sampling procedure

This project was part of a larger study investigating Atlantic sturgeon in the SRE, where a standard protocol of sampling was followed (Kahn and Mohead, 2010). Briefly, sturgeon were removed from the net-pen and the total length, fork length (FL) and head length were measured to the nearest centimetre and the interorbital width to the nearest millimetre. Each Atlantic sturgeon was then scanned with an AVID PowerTracker VIII scanner to check for the presence of an AVID passive integrated transponder (PIT) tag. If no tag was found, a 134.2 kHz PIT tag (model HPT12; Biomark) was inserted into the dorsal musculature at the base of the dorsal fin. Additionally, a US Fish and Wildlife Service T-bar tag was injected opposite the PIT tag in the same location. A 4 ml aliquot of blood was then drawn from the caudal vein using a heparinized 3.17 cm, 22 gauge needle and 7 ml BD Vacutainer tube. The blood samples were stored on ice in the field no longer than an hour until further processing back in the laboratory. After the sampling process commenced, fish were returned to a net-pen and allowed to recover before being released. Finally, in the laboratory, a subsample of each blood sample was analysed for haematocrit (VWR micro-haematocrit capillary tubes), followed by centrifugation at 1242***g*** for 10 min. The plasma was then removed and frozen at −20°C until steroid hormone analysis of circulating levels of testosterone (T) and 17β-estradiol (E_2_) could be completed.

### Steroid hormone extraction

The steroid hormones T and E_2_ were extracted from plasma samples following modified protocols from Sulikowski *et al*. (2016) and Prohaska *et al*. (2013). Briefly, each sample was spiked with 1000 counts min^−1^ of the respective tritiated hormone (Perkin Elmer, Waltham, MA, USA) to account for procedural loss and extracted twice with 10 volumes of ethyl ether (ACS grade). The organic phase was evaporated under nitrogen at 37°C, and each sample was reconstituted in phosphate-buffered saline with 0.1% gelatin. Mean extraction recoveries for T and E_2_ were 78 and 71%, respectively.

### Radioimmunoassay

Plasma concentrations of T and E_2_ were determined by radioimmunoassay (RIA) following a modified protocol by Tsang and Callard (1987). In this protocol, non-radiolabelled T and E_2_ stocks (Steraloids Inc., Newport, RI, USA) were prepared into stock solutions at 80 µg ml^−1^ for T and 6.4 µg ml^−1^ for E_2_ in absolute ethanol (ACS grade). The specifics of the radiolabelled steroids, the antibody characteristics and titres are provided by Sulikowski *et al*. (2004). Radioactivity was detected by a Perkin Elmer Tri-Carb 2900TR liquid scintillation analyser (Waltham, MA, USA). The mean intra-assay coefficients of variance for T and E_2_ were 10 and 9% and the inter-assay coefficients of variance were 11 and 12%, respectively.

### Sex determination

To provide a cross-examination of data in the present study, sex determination by blood hormone concentrations was performed by incorporating findings of two previous studies that used hormones to assess reproductive condition and determine sex and maturity of wild populations (Van Eenennaam *et al*., 1996; Webb *et al*., 2002). For this component of the present study, the mean Atlantic sturgeon androgen (combined values of T and 11-ketotestosterone) and E_2_ concentrations (126.83 ± 62.52 and 0.33 ± 0.15 ng ml^−1^, respectively; values from Van Eenennaam *et al*., 1996) were used as threshold values, but to improve accuracy, the E_2_ concentration was modified to 0.8 ng ml^−1^ (three standard deviations above the reported mean; Van Eenennaam *et al*., 1996). Therefore, an individual in the present study was considered a male if E_2_ concentrations were ≤0.8 ng ml^−1^ or if testosterone concentrations were ≥126 ng ml^−1^. If neither of these conditions was satisfied, the individual was classified as a female.

The second method was a discriminant function analysis by Webb *et al*. (2002) that was generated from white sturgeon (*Acipenser transmontanus*) using T and E_2_ as predicting factors of sex. As such, the following equations from Webb *et al*. (2002) were applied to the SRE Atlantic sturgeon hormone values:

Female = −1.6727 + 2.3678(log_10_T) − −3.5783(log_10_E_2_)

Male = −5.2972 + 5.2524(log_10_T) – 7.5539(log_10_E_2_)

These functions predict the sex of an individual when logarithmically transformed T and E_2_ values are input into each equation. The equation with the higher product then determines the sex (Webb *et al*., 2002). A sturgeon was definitively sexed when both RIA evaluation methods were in accordance, and subsequently, individuals with conflicting sex were categorized as unidentified.

To assess hormonal differences according to sex and size class, mean T and E_2_ were compared between juveniles and adults for each sex (excluding unknown individuals). Owing to non-normality and equal variance within the hormone data, a Kruskal–Wallis one-way ANOVA was performed, followed by Dunn's pairwise multiple comparison. Furthermore, owing to the large number of fish sampled in this study, a sex-predictive model for application to other Atlantic sturgeon populations was modelled. The T and E_2_ values from fish in the known RIA category were fitted in a multiple logistic regression with forward selection (selection criterion = 0.10), with the consequent model predicting the probability of a female Atlantic sturgeon.

A χ^2^ test of homogeneity was used to assess the male-to-female ratio in the SRE of Atlantic sturgeon in the known category. The sex ratio was compared between 2012, 2013 and 2014 to determine whether annual shifts existed. Then, a series of Fisher's exact tests were used to assess the ratio by maturity derived from fork length [for late-stage juvenile (63–134 cm FL) and adult (135–190 cm FL); Bain, 1997] and by the seasonal groupings of spring (April–May), summer (June–August) and autumn (September–November) for each year. The Kruskal–Wallis one-way ANOVA and the multiple logistic regression were analysed using Systat 13 (Systat Software, San Jose, CA, USA), whereas the χ^2^ and Fisher's exact test analyses were performed using R 2.15.2. All statistical analyses were considered significant at α ≤ 0.05.

## Results

The mean haematocrit value from all blood samples was 29 ± 0.5%. Sex was determined for 267 of 288 (93%) Atlantic sturgeon sampled in the present study. When sex determination was assessed by late-stage juveniles (63–134 cm FL) and adults (135–190 cm FL), as defined by Bain (1997), sex was determined in 92 and 97% of Atlantic sturgeon, respectively. Of the individuals with unidentified sex, 67% were classified as juveniles. The mean fork length of unidentified fish was 122 ± 5 cm.

**Figure 1:**
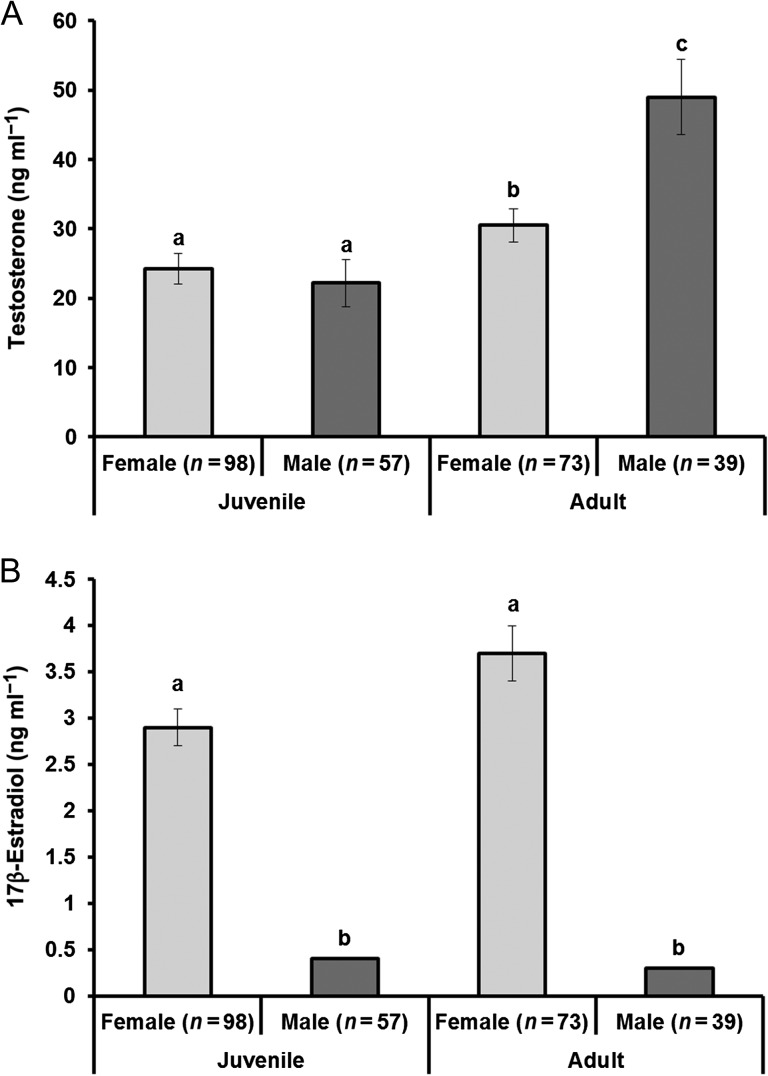
Mean ± SEM testosterone (**A**) and E_2_ (**B**) for late-stage juvenile (63–134 cm fork length) and adult (135–190 cm fork length) Atlantic sturgeon. Means were significantly different at α ≤ 0.05, and are denoted with different letters.

Evaluation of RIA hormone values of T in the Dunn's pairwise comparison revealed that juvenile male and female Atlantic sturgeon were not significantly different from one another (*P* = 1.00; Fig. 1A). However, E_2_ was found to be significantly higher in female juvenile sturgeon than in male juveniles (*P* < 0.001; Fig. 1B). In adult sturgeon, males and females had significantly higher levels of T (*P* = 0.031; Fig. 1A) and E_2_ (*P* < 0.001; Fig. 1B), respectively, over all other groupings. When T concentrations were compared between juvenile and adult sturgeon, the adult mean was significantly higher in males (*P* < 0.001; Fig. 1A) as well as in females (*P* = 0.46; Fig. 1B). The E_2_ concentrations were not significantly different between juvenile and adult females (*P* = 0.054; Fig. 1B) or between juvenile and adult males (*P* = 1.00; Fig. 1B).

Finally, the model fit (d.f. = 2, *P* < 0.0001) from the logistic regression found both T and E_2_ to be significant predictors of Atlantic sturgeon sex in the SRE. The relationship between the two hormones is represented by the following equation:

$$P\left(\mathrm{female}\right)=\frac{e^{-12.9308+\left(16.9520∙E_{2}\right)-(0.0798∙T)}}{{1\;+\;e}^{-12.9308+\left(16.9520{∙E}_{2}\right)-(0.0798∙T)}}$$

When all RIA results from 2012−2014 were pooled, the results indicated an overall sex ratio of 33% males, 60% females, and 7% unidentified sturgeon occurred within the sampled SRE Atlantic sturgeon.

The χ^2^ test of homogeneity of the sex ratio between 2012 and 2013 showed no significant difference (χ^2^ = 5.559, d.f. = 2, *P* = 0.062; Table 1), and also indicated a likely 1:1 ratio (male:female; 2012 and 2013, 41% male, 50% female and 9% unidentified). However, 2012 and 2013 each showed a significant shift in sex ratio in comparison to 2014 (2012, χ^2^ = 9.423, d.f. = 2, *P* = 0.009; and 2013, χ^2^ = 14.896, d.f. = 2, *P* = 0.001; Table 1) attributable to an increase in the number of females present in the SRE (2014, 23% male, 72% female and 4% unidentified). No significant difference between the overall sex ratio for juvenile and adult Atlantic sturgeon for any sampling year was observed via Fisher's exact test (2012, *P* = 0.808; 2013, *P* = 1.000; and 2014, *P* = 0.682; Table 2), indicating that the female skewed ratio in 2014 existed for both juveniles and adults alike.

**Table 1:** Counts and percentages of males, females and unidentified Atlantic sturgeon for the entirety of the study and for individual years

Sex	Total		2012^a^		2013^b^		2014^a,b^	
	*n*	Percentage	*n*	Percentage	*n*	Percentage	*n*	Percentage
Male	96	33.5	30	44.9	36	38.7	30	23.4
Female	171	59.6	35	52.2	44	47.3	92	71.9
Unidentified	21	6.9	2	2.9	13	14.0	6	3.9

Letters represent statistical differences in sex ratios compared by χ^2^ contingency tables.

**Table 2:** Counts and percentages of males, females and unidentified Atlantic sturgeon for late-stage juvenile (63–134 cm fork length) and adult (135–190 cm fork length) sturgeon sampled each year

	Juvenile		Adult	
	*n*	Percentage	*n*	Percentage
A. 2012				
Male	14	41.2	16	48.5
Female	19	55.9	16	48.5
Unidentified	1	2.9	1	3.0
B. 2013				
Male	25	39.0	11	37.9
Female	30	46.9	14	48.3
Unidentified	9	14.1	4	13.8
C. 2014				
Male	18	25.4	12	21.1
Female	49	69.0	43	75.4
Unidentified	4	5.6	2	3.5

No statistically significant differences were found between juvenile and adult percentages in any given year.

The analysis by season indicated that the maturity and sex ratio did not change throughout the sampling in 2012 and 2014 (Table 3A and C). However, although the overall proportion of male to female was the same between juveniles and adults in 2013 (Table 2B), there was a significant shift from summer to autumn in the ratio (*P* = 0.002; Table 3B). Owing to the low samples size (*n* = 9), data from the spring could not be evaluated in any of the sampling years (Table 3A, B and C).

**Table 3:** Counts and percentages of males, females and unidentified Atlantic sturgeon for seasons from all years sampled

Sex	Maturity	Spring*		Summer^a^		Autumn^b^	
		*n*	Percentage	*n*	Percentage	*n*	Percentage
A. 2012							
Female	Juvenile	0	0	15	37.5	4	19.1
	Adult	0	0	9	22.5	7	33.3
Male	Juvenile	0	0	8	20.0	6	28.6
	Adult	4	100.0	8	20.0	4	19.0
B. 2013							
Female	Juvenile	0	0	26	44.1	4	20.0
	Adult	0	0	11	18.6	3	15.0
Male	Juvenile	1	100.0	19	32.2	5	25.0
	Adult	0	0	3	5.1	8	40.0
C. 2014							
Female	Juvenile	1	25.0	35	39.8	13	43.3
	Adult	1	25.0	32	36.4	10	33.3
Male	Juvenile	2	50.0	15	17.0	1	3.4
	Adult	0	0	6	6.8	6	20.0

A significant difference was found only between summer and autumn of 2013 using Fisher's exact test and is denoted by differing letters. *Low sample size in April–May, precluding a statistical test of the spring category.

## Discussion

The present study used a non-lethal approach to determine the sex, reproductive status and the overall sex ratio of Atlantic sturgeon in the SRE. By using two methods to assess the proportion of T and E_2_ in each fish, this study was effective at identifying sex in 93% of all sampled Atlantic sturgeon. Furthermore, the 92 and 97% effectiveness of the method in juveniles and adults, respectively, indicates that this method is valuable over multiple life stages in wild fish. Although it was hypothesized that the majority of unidentified fish would be juvenile owing to pre-maturation hormone concentrations, the mean fork length of 121 ± 0.5 cm reveals that size was not a likely factor in sex determination.

Testosterone concentrations in this study could not be directly compared with Atlantic sturgeon in the Hudson River because Van Eenennaam *et al*. (1996) assessed total androgen concentrations (including 11-ketotestosterone). In comparison to T concentrations in male maturing white sturgeon (150 ng ml^−1^), the mean SRE sturgeon value (49 ± 5.4 ng ml^−1^) was lower (Webb *et al*., 2002). However, the adult male T mean in the SRE was higher than means in lake sturgeon (Craig *et al*., 2009) and ‘the bester’ [F_1_ hybrid beluga and sterlet sturgeon (*Huso huso* L. female × *Acipenser ruthenus* L. male); Amiri *et al*., 1996a], where T peaked at 5.023 and 28 ng ml^−1^, respectively in each species.

The present study found that adult females had the highest mean estradiol levels of all categories assessed, which has also been observed in Atlantic sturgeon in the Hudson River (Van Eenennaam *et al*., 1996). The mean value of E_2_ in SRE Atlantic sturgeon (3.7 ± 0.3 ng ml^−1^) was similar to that of ovulating (1.99 ± 1.32 ng ml^−1^) and gravid female (5.10 ± 4.05 ng ml^−1^) means of Atlantic sturgeon in the Hudson River (Van Eenennaam *et al*., 1996), indicating that some SRE sturgeon may be undergoing vitellogenesis. This trend also corresponds to findings in maturing female white sturgeon (Webb *et al*., 2002), showing the utility of a sex-determining equation between species, as used in the present study. The mean E_2_ concentration in the present study (3.7 ± 0.3 ng ml^−1^) fell within the range of female adult ‘the bester’ hybrid (2–4 ng ml^−1^; Amiri *et al*., 1996b) and female lake sturgeon (0.025–9.536 ng ml^−1^; Craig *et al*., 2009) but was lower than adult female Gulf sturgeon (mean, 9.97 ng ml^−1^; Heise *et al*., 2009). Additionally, in the present study, the immature female E_2_ mean was an intermediate value (2.9 ± 0.2 ng ml^−1^) between mature females and all male sturgeon. This finding was unique to the present study, whereas Webb *et al*. (2002) found that immature female white sturgeon had E_2_ concentrations with no significant difference from male immature and maturing white sturgeon.

The innate differences in T and E_2_ levels between the sexes illustrates the utility of circulating steroid hormones as a non-lethal method for sex determination of Atlantic sturgeon. However, the lack of significant differences of hormone values between juveniles and adults of each sex was unexpected. Juvenile individuals would be expected to have lower basal levels of T and E_2_, and conversely, adults would have significantly higher levels (e.g. Webb *et al*., 2002; Craig *et al*., 2009; Petochi *et al*., 2011). These high levels of T and E_2_ in the SRE could be attributed to individuals that are considered late-stage juvenile by FL that were undergoing sexual maturation at the time of sampling. The gonadal changes associated with maturation could manifest as higher circulating levels of reproductive hormones in the blood.

The logistic model created in the present study provides the first species-specific method of sex determination with hormones for both juvenile and adult Atlantic sturgeon, providing a new tool for future studies using RIA. The classification used in this study from Webb *et al*. (2002) has been successfully used in lake sturgeon (*Acipenser fulvescens*), showing the utility of these types of classification equations even across sturgeon species (Craig *et al*., 2009; Shaw *et al*., 2012; Thiem *et al*., 2013). However, the model created herein needs further investigation with spawning populations, in which mean hormone concentrations are generally higher than the mean values of T and E_2_ in the SRE (Van Eenennaam e*t al.*, 1996). It is important to note that in the present study we could not validate sex by dissection or endoscopy because of the endangered status of the species. However, the combination of sex-determining methods from Webb *et al*. (2002) and Van Eenennaam e*t al.* (1996) allows for a high degree of accuracy in the findings.

A unique and unexpected finding of the present study was a shift to a female-dominated sex ratio that was observed in 2014, as non-spawning populations usually maintain a near 1:1 (male:female) sex ratio (Trencia *et al.,* 2002). Therefore, a female-biased sex ratio of 1:3 (male:female) observed in a non-spawning system, such as the SRE, is uncommon. For example, in the St Lawrence River, Atlantic sturgeon have been found in varying sex ratios depending on the area, but all were relatively balanced, ranging from 1:1.13 to 1:1.32 (male:female) during the non-spawning seasons (Trencia *et al*., 2002). Likewise, in a shovelnose sturgeon (*Scaphirhynchus platorynchus*) population, the total sex ratio was 1.2:1 (male:female), but shifted to 2.3:1 when only spawning individuals were considered (Moos, 1978).

Although sex ratios by season had a small sample size (*n* = 9), precluding a statistical test, more male Atlantic sturgeon were observed during April and May. Atlantic sturgeon in the SRE enter the system in mid-spring (Little 2013), with results from the present study probably substantiating that a male cohort is the first to aggregate (Table 3). This finding is not uncommon as male-dominated sex ratios have been documented in other sturgeon species. For example, in shortnose sturgeon (*Acipenser brevirostrum*), during spawning aggregations sex ratios have been documented ranging from 2.5:1 to 7:1 (male:female) in various systems (Pekovitch, 1979; Taubert, 1980a, b; Buckley and Kynard, 1985b; Collins and Smith, 1997). Likewise, in lake sturgeon (*Acipenser fulvescens*) a ratio of 2.1:1 (male:female) was observed in a spawning aggregation (Thiem *et al*., 2013). However, unlike male sturgeon in other systems, the male-skewed sex ratio is probably not a product of a spawning aggregation, and further investigation of this observation on a large temporal scale would help to elucidate whether the observed skewed sex ratio has ecological or biological significance.

Although the SRE cannot be used as a spawning ground owing to lack of assess by unpassable dams, preliminary research suggests it may serve as a foraging site (Little, 2013). Sand lance (*Ammodytes americanus*) comprise the majority (Percent Index of Relative Importance = 83.4%) of the Atlantic sturgeon diet in the SRE, which has not been documented elsewhere. The high nutrient content of Atlantic sand lance could be beneficial energy for growth of juveniles and future reproduction in adults, in which nutrient-rich food sources can increase fecundity and egg viability (Ouellet *et al*., 2001; Cerdá *et al*., 2004). However, more research is needed in this area to determine whether foraging in the SRE affects energy stores (i.e. fatty acids) that in turn could affect Atlantic sturgeon population health outside of this small estuarine system.

In summary, the present study used a non-lethal approach to determine the sex, reproductive status and the overall sex ratio of Atlantic sturgeon in the SRE. By doing so, the present investigation illustrates the need to gain a better understanding of the importance of small estuaries to the GOM DPS unit and the Atlantic sturgeon population as a whole. For future conservation of this species, non-lethal methods should be applied to other understudied river systems to complete a larger data set of Atlantic sturgeon throughout their range.
